# Biomarkers of *Plasmodium falciparum* Infection during Pregnancy in Women Living in Northeastern Tanzania

**DOI:** 10.1371/journal.pone.0048763

**Published:** 2012-11-14

**Authors:** Stéphanie Boström, Samad Ibitokou, Mayke Oesterholt, Christentze Schmiegelow, Jan-Olov Persson, Daniel Minja, John Lusingu, Martha Lemnge, Nadine Fievet, Philippe Deloron, Adrian J. F. Luty, Marita Troye-Blomberg

**Affiliations:** 1 Department of Immunology, Wenner-Gren Institute, Stockholm University, Stockholm, Sweden; 2 Centre d’étude et de recherche sur le paludisme associé à la grossesse et à l’enfance (CERPAGE), Faculté des Sciences de la Santé, Cotonou, Benin; 3 Faculté de Pharmacie, Université Paris Descartes, Sorbonne Paris Cité, Paris, France; 4 Department of Medical Microbiology, Radboud University Nijmegen Medical Centre, Nijmegen, The Netherlands; 5 Centre for Medical Parasitology, Department of International Health, Immunology and Microbiology, University of Copenhagen and Department of Infectious Diseases, Copenhagen University Hospital, Copenhagen, Denmark; 6 Division of Mathematical Statistics, Department of Mathematics, Stockholm University, Stockholm, Sweden; 7 National Institute for Medical Research, Tanga, Tanzania; 8 Institut de Recherche pour le Développement (IRD) UMR216, Tropical Infections in Mother and Child, Paris, France; Liverpool School of Tropical Medicine, United Kingdom

## Abstract

In pregnant women, *Plasmodium falciparum* infections are an important cause of maternal morbidity as well as fetal and neonatal mortality. Erythrocytes infected by these malaria-causing parasites accumulate through adhesive interactions in placental intervillous spaces, thus evading detection in peripheral blood smears. Sequestered infected erythrocytes induce inflammation, offering the possibility of detecting inflammatory mediators in peripheral blood that could act as biomarkers of placental infection. In a longitudinal, prospective study in Tanzania, we quantified a range of different cytokines, chemokines and angiogenic factors in peripheral plasma samples, taken on multiple sequential occasions during pregnancy up to and including delivery, from *P. falciparum*-infected women and matched uninfected controls. The results show that during healthy, uninfected pregnancies the levels of most of the panel of molecules we measured were largely unchanged except at delivery. In women with *P. falciparum*, however, both comparative and longitudinal assessments consistently showed that the levels of IL-10 and IP-10 increased significantly whilst that of RANTES decreased significantly, regardless of gestational age at the time the infection was detected. ROC curve analysis indicated that a combination of increased IL-10 and IP-10 levels and decreased RANTES levels might be predictive of *P. falciparum* infections. In conclusion, our data suggest that host biomarkers in peripheral blood may represent useful diagnostic markers of *P. falciparum* infection during pregnancy, but placental histology results would need to be included to verify these findings.

## Introduction

*Plasmodium falciparum* infections during pregnancy cause substantial maternal and neonatal morbidity and mortality [1]. Malaria during pregnancy, often referred to as placental malaria (PM), results from infected erythrocytes (iE) binding to chondroitin-sulphate A (CSA) in the placenta [2]. A major consequence of PM is low birth weight (LBW) [3]. The reason for this is not completely understood but may be due to impaired uteroplacental blood flow [4], metabolic/growth hormone disturbances [5], alterations of the syncytiotrophoblast layer [6], or impaired trophoblast invasion, leading to intrauterine growth retardation. This makes prompt and accurate diagnosis of PM extremely important, but the combination of the asymptomatic character of infections and the frequent paucity of iE in peripheral blood smears makes diagnosis difficult. In a recent study from Malawi [7], the authors investigated whether a different suite of biomarkers could predict placental infection at delivery in the absence of circulating parasites. The results from that study suggested that host biomarkers in peripheral blood may indeed improve the detection of PM when parasites are undetected in circulation.

PM is usually more frequent and more severe in primigravidae as they lack antibodies that inhibit iE binding to CSA. Sequestration of iE in intervillous spaces leads to monocytic inflammatory infiltration in the placenta [8], [9]. This inflammation may affect cellular functions by altering the cytokine and chemokine balance both in the periphery and in the placental blood [10]–[15]. Fetal and maternal cells secrete inflammatory and immunoregulatory molecules in response to sequestered iE [11]. In this context it is notable that PM and pre-eclampsia share many features including an altered cytokine balance and some studies have demonstrated an increased risk of preeclampsia among pregnant women with malaria [16].

Pregnancy represents a state of immunological tolerance in which maternal pro-inflammatory T helper (Th) lymphocyte type 1 cytokines are down-regulated to protect the fetus from allograft rejection [17]. Th1-type responses in the placenta are detrimental for the fetus: high levels of pro-inflammatory cytokines have been shown to be incompatible with successful pregnancy in mice [18]. In humans, PM results in elevated levels of both tumor necrosis factor (TNF)-α and interferon (IFN)-γ in placental plasma, affecting the delicate cytokine balance [12], [13]. These cytokines may help eliminate parasites by enhancing the phagocytic activity of monocytes/macrophages, but uncontrolled inflammatory responses in the placenta could be pathological, interfering with normal maternal-fetal exchange. Chemokines mediate the initial inflammatory responses to pathogens via chemotactic interactions with their corresponding receptors expressed on multiple leucocyte cell-types. Binding of iE in the placenta leads to chemokine secretion that stimulates leucocyte infiltration and initiates an inflammatory cascade. Several chemokines are increased during PM [11], some associated closely with monocytic infiltrates [10].

A recent study from Cameroon [19] reported an association between plasma soluble TNF receptor-2 levels and LBW in women infected by *P. falciparum*, suggesting that biomarkers in peripheral blood might discriminate women with poor pregnancy outcomes as a function of malarial infection status. Since PM induces a local host response in the placenta, and soluble components from the placental compartment may circulate in the peripheral blood, investigating host proteins as possible candidate biomarkers might be a good way to detect PM. In the study described here peripheral venous plasma concentrations of several pro- and anti-inflammatory molecules and angiogenic factors were measured on multiple occasions during pregnancy and at delivery in a cohort of Tanzanian women, and their association with infection by *P. falciparum* was evaluated.

## Methods

### Ethics Statement

Written informed consent was obtained from all mothers before inclusion, and ethical clearance was obtained from the Tanzanian Medical Research Coordinating Committee (NIMR7HQ/R.8a/Vol.IX/688).

### Study Area

This study was carried out between September 2008 and October 2010 in the Korogwe district, located about 100 kilometers inland from the coastal city of Tanga, northeastern Tanzania. Historically, malaria transmission in the area was reported to be intense and perennial but with seasonal peaks during and following the rainy seasons from March to July and from October to December [20]. However, malaria transmission has markedly declined in recent years [21]. *P. falciparum* is the predominant malaria species in the area [21].

### Study Design

STOPPAM (“Strategies To Prevent Pregnancy Associated Malaria”), a longitudinal cohort study of pregnant women, was conducted in parallel in two separate sites in Tanzania and Benin. In both study sites, 1000 pregnant women with a gestational age ≤24 weeks based on ultrasound evaluation were included and followed during pregnancy with a series of scheduled antenatal visits (ANV) until delivery. Delivery samples were collected within 24–48 hours of delivery. Details of the study design and procedures used have been published elsewhere [22]. Here we present data from a part of the study focused on immunological aspects conducted in the Tanzanian study site.

### Parasitological Diagnoses and Treatment

For diagnosis of plasmodial infection at each ANV and at delivery, Parascreen™ (Zephyr Biomedical Systems) rapid diagnostic tests (RDT) were used except during May-July 2009 and July-September 2009 when ParacheckPf® (Orchid Biomedical Systems) and ParaHIT®f (Span diagnostics Ltd) were used, respectively. Thick and thin blood smears were also systematically made at each visit, as well as placental impression smears at delivery. Smears were routinely stained with Giemsa and read by two expert microscopists. PCR-based detection was not used. All women presenting with infection diagnosed by RDT at any ANV received anti-malarial treatment according to the national guidelines.

### Study Population

For the sub-study described here a group of 121 pregnant women (42 infected and 79 uninfected) was retrospectively identified for the assessment of potential bio-markers in peripheral venous plasma. The infected women were selected based on the following criteria: (i) *P. falciparum* infection - defined by the combination of a positive RDT and the presence of parasites in blood/placental impression smears - once during pregnancy, (ii) attendance at all three scheduled ANV at gestational ages 26 (ANV2), 30 (ANV3), and 36 (ANV4) and at delivery, with corresponding plasma samples available and (iii) being HIV seronegative and not pre-eclamptic. Each infected woman was matched to two separate uninfected controls of similar age (±4 years), gestational age (±2 weeks) at the time the infection was detected, and gravidity. Of note, of the 1000 pregnant women enrolled 78 were identified at some point during pregnancy to be infected with *Plasmodium*. Of these 78 women, 42 met the above mentioned criteria to be included in this sub-group for analysis of biomarkers. The characteristics of these pregnant women are summarized in Table 1.

**Table 1:** Description of the pregnant women included in the present study.

Characteristics	Uninfected	Infected	*p*
**Number of subjects (n)**	79	42	
**Age of mother, (mean ± SD), years**	24±5.3	24±5.3	
**Mean parasitemia, (min-max ± SD), (parasites/µl)**	-	27969.2 (39.5–390749±17132.4)	
**Primigravidae**	22	12	
**Secundigravidae**	33	17	
**Multigravidae ≥3**	25	13	
**Neonatal birth weight (median ± IQR), g**	3200±600	3000±780	0.067^b^

b Mann-Whitney non-parametric U test.

### Sample Collection

Venous blood samples from the women were collected at all visits in vacutainers (Greiner bio-one, Denmark) with citrate phosphate dextrose adenine anticoagulant. After centrifugation, undiluted plasma was collected, aliquoted and stored at −80°C until use in assays.

### Cytometric Bead Arrays

Levels of IL-1β, IL-6, IL-8, IL-10, IL-12p70, TNF, regulated on activation normal T cell expressed and secreted (RANTES), monokine-induced by IFN-γ (MIG), monocytes chemotactic protein (MCP)-1 and IFN-gamma-inducible protein (IP)-10 were measured in plasma using cytometric bead arrays (CBA, BD Biosciences, San Diego, CA, USA) according to the manufacturer’s recommendations. The samples and standards were acquired on a flow cytometer (FACSCalibur, Becton Dickinson, France) and analyzed using FCAP Array software v1.0.1 (BD/Softflow, Hungary). Calibration was performed on the flow cytometer before acquisition using BD FACSComp™ and BD CaliBRITE™ beads. The lower detection limits were 3.6, 7.2, 2.5, 3.3, 3.7, 1.9, 1.0, 2.5, 2.7 and 2.8 pg/ml for IL-8, IL-1β, IL-6, IL-10, TNF, IL-12p70, RANTES, MIG, MCP-1 and IP-10, respectively. All samples from a given woman were assayed simultaneously and the positive women were always assayed together with the corresponding control women’s plasma samples.

### ELISA

Commercially available ELISA-based kits for IFN-α, IFN-γ (Mabtech, Stockholm, Sweden), vascular endothelial growth factor receptor 1 (VEGF R1/Flt1), urokinase receptor (uPAR), Angiopoietin (Ang)-1 and Ang-2 (R&D system, Minneapolis, MN) were used according to the manufacturer’s recommendations. The enzyme-substrate reaction was developed using p-nitrophenyl phosphatase (Sigma, St Louis, MO, USA**)** for IFN-α and IFN-γ and tetramethylbenzidine substrate (R&D systems) for the others, measuring optical densities in a multiscan ELISA reader at 405 and 450 nm, respectively. The concentrations were calculated from standard curves established with corresponding purified recombinant human proteins. The lower detection limits were 7, 2, 78, 16, 27, 78 pg/ml for IFN-α, IFN-γ, VEGF R1/Flt1, uPAR, Ang-1 and Ang-2, respectively.

### Statistical Analysis

Statistical differences in plasma protein concentrations between *P. falciparum* infected women and controls were evaluated by the Mann-Whitney U non-parametric test. Statistical significance was declared when p<0.05. To detect significant changes in plasma protein concentrations during healthy pregnancies, Friedman’s test was performed with observations from all five time points for the non-infected women. Multiple logistic regression was used to assess the association between different molecules and *P. falciparum* infection. A stepwise procedure was performed to select a model including the factors with the strongest association with infection. The predictive power of such a model was summarized using receiver operating characteristics (ROC) curves, and area under ROC curve (AUC). The observations used for these analyses were from the time point for infection together with the corresponding matched observations. The statistical software packages used were StatView 5.0.1 and Stata 12.

## Results

### Cytokines, Chemokines and Other Factors During Pregnancy in Plasma Obtained from *P. falciparum* Negative Pregnant Women

**Figure 1:**
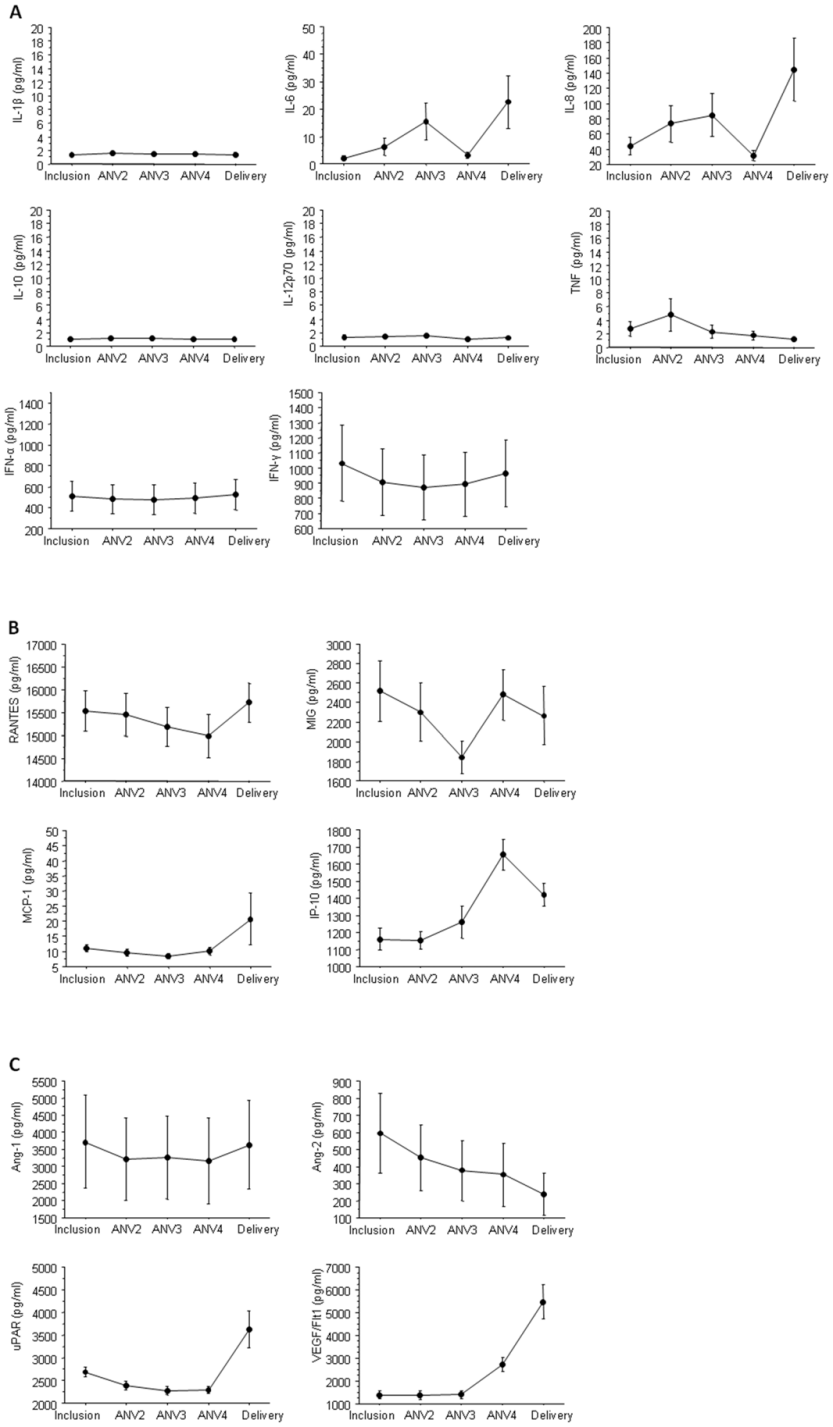
Baseline plasma levels of several inflammatory factors and others throughout pregnancies not complicated by malaria. Plasma levels of (A) cytokines, (B) chemokines, and (C) angiogenic factors in plasma samples from 79 uninfected pregnant women measured throughout their pregnancy. Samples were analyzed at inclusion, at antenatal visit (ANV) 2, 3 and 4 and at delivery by ELISA or CBA. The dots represents the mean value and the bars the standard deviation.

Little is known about cytokine and chemokine levels during normal pregnancies and to our knowledge baseline levels at different gestational ages of African populations have not been reported. We therefore first determined the levels of a panel of markers (IL-1β, IL-6, IL-8, IL-10, IL-12p70, TNF, RANTES, MIG, MCP-1, IP-10, IFN-α, IFN-γ, Ang-1, Ang-2, uPAR and VEGF R1/Flt1) in the plasmas of 79 women who remained infection-free from inclusion through to delivery (Fig. 1). The levels of the majority of the cytokines (Fig. 1A), chemokines (Fig. 1B) and angiogenic factors other factors (Fig. 1C) were not statistically significantly different over time with the exception, at delivery, of IL-6, IL-8, IP-10, uPAR and VEGF R1/Flt1 that increased notably (p<0.0001 for all). TNF and IL-1β were in most cases undetectable (Fig. 1A).

### Inflammatory Factors and *P. falciparum* Infection

**Figure 2:**
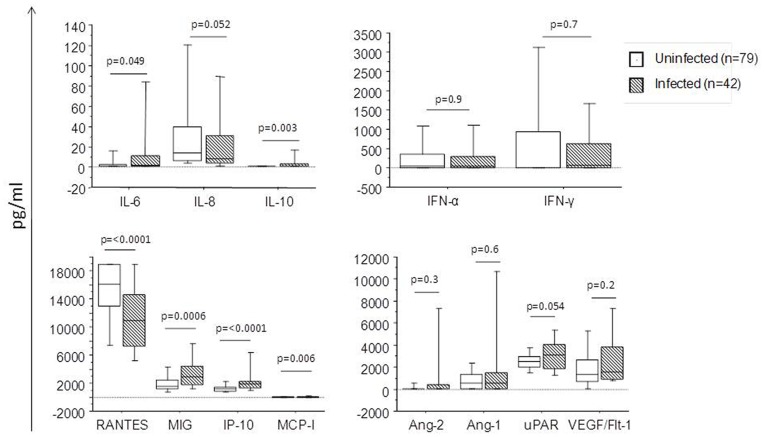
Plasma levels of several inflammatory and other factors in pregnant women with or without *P. falciparum* infection. Plasma levels of selected factors were measured in 42 *P. falciparum* infected women, who were infected once at single time points during pregnancy, and matched to 79 uninfected controls. The factors were measured using ELISA or CBA. The boxes represent the values between 25% and 75% quartile and the line indicates the median. The whiskers indicate the 10% and 90% percentiles. The *p*-values were determined by non-parametric Mann-Whitney U test.

To evaluate the effect of *P. falciparum* infection we compared the concentrations of the different molecules in the plasmas of infected and uninfected pregnant women (Fig. 2). The level of RANTES was significantly lower whilst the levels of IL-6, IL-10, MIG, MCP-1 and IP-10 were all significantly higher in the infected women. Levels of IL-8 and uPAR were also altered as a result of infection, but in neither case did the difference reach statistical significance. The levels of Ang-1, Ang-2, IFN-α, IFN-γ and VEGF R1/Flt1 were unaffected by infection (Fig. 2). Of note, there were no differences at any time in the levels of either TNF or IL-1β between infected and uninfected individuals (data not shown).

### Longitudinal Assessment of IL-10 and IP-10 during Pregnancy

**Figure 3:**
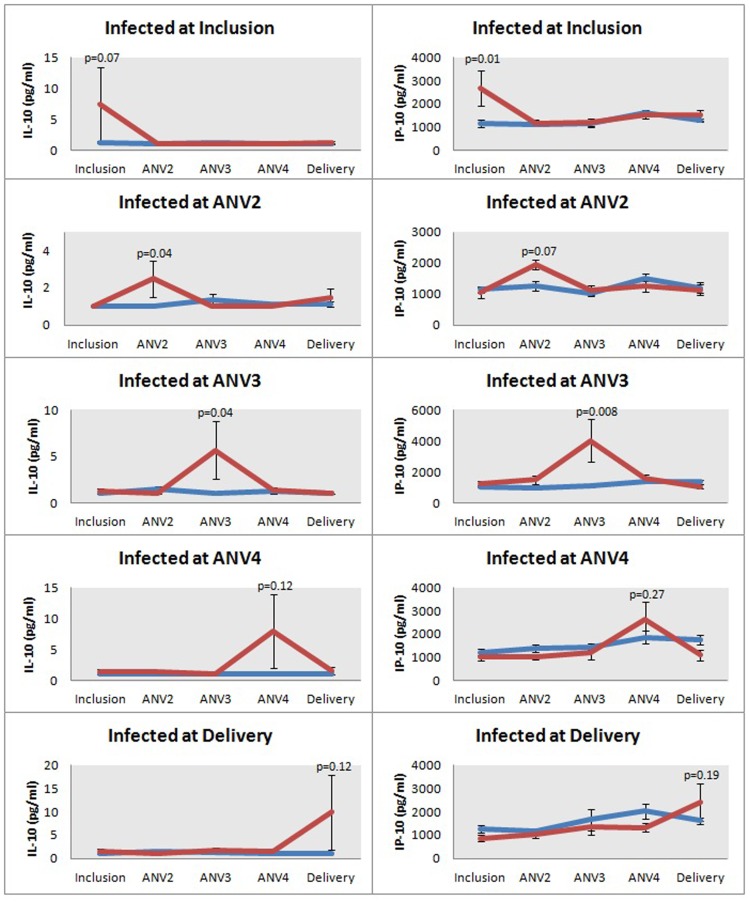
Longitudinal assessment of IL-10 and IP-10 during pregnancy. All women that were infected during their pregnancy were grouped according to the time point when the women were infected. For each group of infected women, samples from the negative control women at the same occasion were grouped together in the same graph. The three antenatal visits (ANV) were at gestational ages 26 (ANV2), 30 (ANV3), and 36 (ANV4). At inclusion: uninfected (n = 24), infected (n = 13); at ANV2: uninfected (n = 13), infected (n = 7); at ANV3: uninfected (n = 15), infected (n = 8); at ANV4: uninfected (n = 13), infected (n = 7); at Delivery: uninfected (n = 14), infected (n = 7). The red lines illustrate mean values for the infected women and the blue line illustrates the mean values for the uninfected control women. The statistical significance of differences in the concentrations between infected and uninfected women at the different time-points is illustrated.

We next determined whether the levels of the different molecules changed as a function of women’s gestational age at the time of infection with *P. falciparum*. For this purpose, the infected and matched uninfected women were grouped according to the gestational age at the time infection was identified (Fig. 3). The results showed that the levels of both IL-10 and IP-10 increased significantly when women were infected, irrespective of gestational age. Following anti-malarial treatment the levels decreased to background levels as reflected consistently by the assessments of samples taken at the subsequent ANV. Of all the molecules evaluated in this way, only IL-10 and IP-10 showed this consistent infection/treatment-related change in profile (Fig. 3 and data not shown).

### Levels of Markers Differ Based on Gravidity and Infection Status

**Figure 4:**
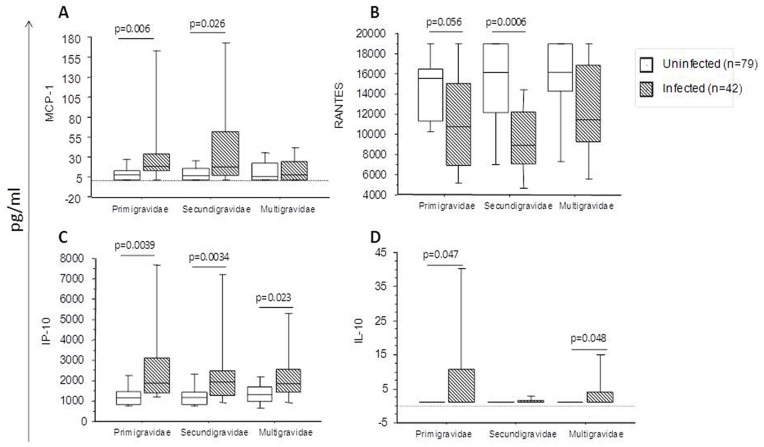
Comparison of inflammatory factors in peripheral plasma from *P. falciparum* infected and uninfected women stratified by gravidity status. Plasma levels of (A) MCP-1 (B) RANTES, (C) IP-10 and (D) IL-10 according to gravidity of the women. Infected primigravidae (n = 12), uninfected primigravidae (n = 22), infected secundigravidae (n = 17), uninfected secundigravidae (n = 33), infected multigravidae (n = 13) and uninfected multigravidae (n = 25). The boxes represent the values between 25% and 75% quartile and the line indicates the median. The whiskers indicate the 10% and 90% percentiles. *P*-values were determined by non-parametric Mann-Whitney U test.

Primigravidae are at greatest risk of infection with *P. falciparum* and are more likely to suffer severe complications and to have poorer pregnancy outcomes compared to multigravidae. We therefore assessed the levels of different factors according to gravidity (Fig. 4). Infected primigravidae and secundigravidae had significantly higher levels of MCP-1 (Fig. 4A) but lower levels of RANTES (Fig. 4B) compared to their uninfected counterparts. These differences were not seen in the multigravidae, although RANTES displayed the same trend towards lower concentrations in infected women. Levels of IP-10 were significantly higher in all infected women, irrespective of gravidity, compared to uninfected women (Fig. 4C). The levels of IL-10 were significantly increased in infected primigravidae and multigravidae but not in secundigravidae (Fig. 4D).

### Predicting *P. falciparum* Infection during Pregnancy Associated Malaria

**Figure 5:**
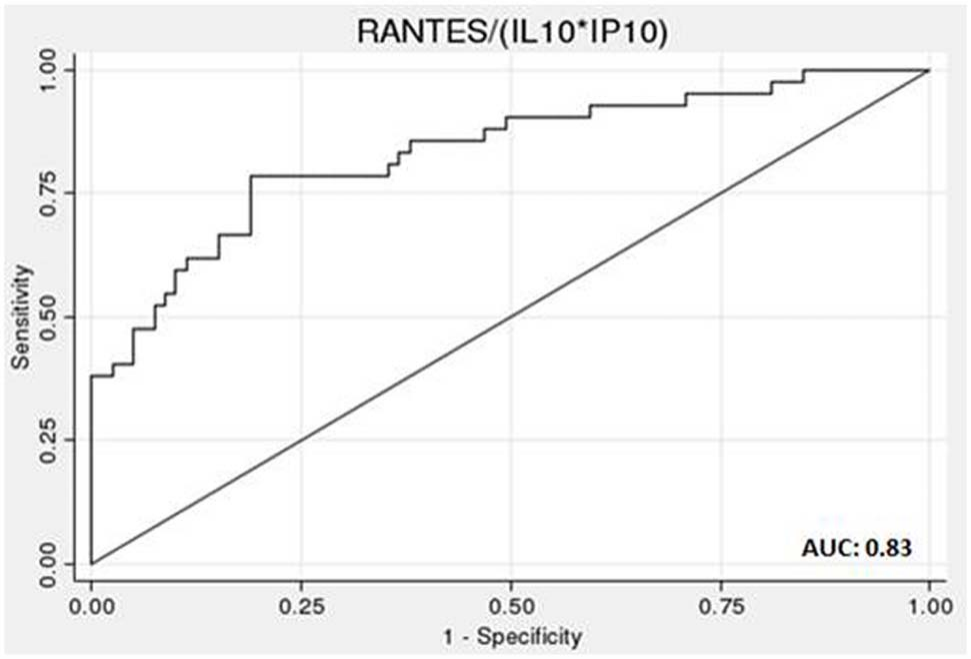
Assessment of biomarkers in predicting *P. falciparum* infection during pregnancy. Receiver operating curve (ROC) for the multiple logistic regression model, with IL-10, IP-10 and RANTES as predictors. The area under the ROC curve (AUC) with 95% confidence interval is 0.83 (0.75, 0.91).

We included a number of biomarkers in our analysis that have previously been shown to be altered in the peripheral blood of women with placental malaria. To investigate their predictive value for infection, selected factors were analyzed using multiple logistic regression (Table 2). The results show that the likelihood of having been infected with *P. falciparum* increases by a factor of 2.85, 2.82 and 0.32 with the doubling of concentration of IP-10, IL-10 and RANTES, respectively. We further analyzed the diagnostic accuracy of these three putative biomarkers using ROC curve analysis (Fig. 5). Individually all markers displayed moderate predictive ability with areas under the curve (AUC) between 0.61–0.77 (data not shown). However, when combining the factors identified by the multiple logistic regression analyses, we found the combination of elevated IL-10 and IP-10 levels with decreased RANTES levels to be predictive of infection, with the highest AUC of 0.83 (Fig. 5).

**Table 2:** Estimated odds ratio with 95% confidence interval for the multiple logistic regression model.

Factor	Odds ratio	95% CI	*p*
**IP-10**	2.85	1.39–5.84	0.004
**IL-10**	2.82	1.21–6.58	0.016
**RANTES**	0.32	0.15–0.68	0.004

Concentration values of factor 2 were log2-transformed which means that odds ratio estimate applies for a doubling of concentration.

## Discussion

An essential component of the sub-study described here comprised the detailed longitudinal clinical and parasitological surveillance of women during pregnancy that was an integral aspect of the overall STOPPAM study. This enabled us to make assessments of sequential samples from a sub-group of women in whom a single defined asymptomatic infection with *P. falciparum* was identified, along with equivalent samples from appropriately matched women who remained infection-free up to and including delivery. The study design thus sets it apart from other published studies. Amongst the latter, those that focus on individuals with asymptomatic *P. falciparum* are scarce. Children with asymptomatic *P. falciparum* infections have elevated plasma IFN-γ, TNF and IL-4 levels [23], whilst a cross-sectional study of third trimester pregnancies reported increased G-CSF and IL-10 in women with asymptomatic infections [24]. To our knowledge, few studies have used a longitudinal design to assess aspects of the timing of infection during pregnancy and the relationship with potential biomarkers in plasma. The advantage of using such a prospective study design is that it gives a full picture of changes in the levels of such biomarkers as a function both of gestational age and of a defined infection event rather than the ‘snapshot’ image that a cross-sectional design gives. For example, a longitudinal study involving a relatively small number of women in Cameroon identified changes in plasma angiopoietin levels during pregnancy as a function of infection with *P. falciparum* [25], [26]. Such infections were shown by Thévenon and colleagues – using samples from the same study in Cameroon – to be associated with altered TNF receptor profiles [19]. Importantly, we have shown that, over the course of a normal pregnancy, the concentrations of the majority of the factors measured remained stable with few fluctuations except at delivery. Notably, however, at times when the women were infected with *P. falciparum* the levels of several molecules changed, in particular those of IL-6, IL-10, MIG, MCP-1 and IP-10 that increased, while the level of RANTES decreased. Integral to our study design was the fact that the comparisons made were with the levels of the same factors in two uninfected women, who were matched for several potentially confounding parameters. Consistently increased concentrations in relation to infection, regardless of gestational age, were only seen for IL-10 and IP-10. The latter two factors were also found to be present at significantly higher concentrations in infected primigravidae, the group known to be most susceptible to malaria during pregnancy. ROC curve analyses revealed the combination of IL-10, IP-10 and RANTES to be strongly predictive of *P. falciparum* infection during pregnancy.

The fact that the levels of most factors studied were stable, from inclusion right through to delivery, indicates a strict degree of regulation during pregnancy. In particular, the levels of IFN-α and IFN-γ, factors known to be associated with spontaneous abortions and preterm delivery [18] were very stable. Notable also was the absence of detectable levels of TNF in the peripheral plasma of both uninfected and infected women. Numerous studies have shown TNF to be elevated in placental plasma during *P. falciparum* infection [12], [13], [15]. This again suggests tight regulation restricting potentially harmful molecules to isolated areas such as the placenta. Emphasizing the latter, one of those studies did quantify TNF levels in a small number of peripheral plasma samples at delivery and found that the relatively low concentrations detected were weakly associated with *P. falciparum* infection [15]. Of note, other factors measured in our study, especially IL-6, IL-8, IP-10, uPAR and VEGF R1/Flt1, increased markedly at delivery. These findings are consistent with those reported by others [27], showing increased IL-6 levels at the end of normal pregnancies, possibly contributing to the process of initiation of labor. Increased levels of IL-6 at delivery have been reported in pre-eclampsia [28], indicating that IL-6 can be harmful, possibly playing a role in the inflammation and endothelial dysfunction associated with pre-eclampsia.

That infection with *P. falciparum* during pregnancy alters the cytokine balance in both placenta and periphery is widely accepted. We consequently found elevated levels of MIG, MCP-1 and IP-10 in infected women compared to uninfected controls. MIG, MCP-1 and IP-10 are all chemokines that attract different immune cell populations to the sites of infection. MIG and IP-10 are α (CXC) chemokines that are produced by a variety of leucocytes in response to IFN-γ and TNF and are chemoattractants for activated T and NK cells and macrophages. MCP-1 is a β (CC) chemokine produced primarily by monocytes, macrophages and endothelial cells and is a potent monocyte chemoattractant [29]. Monocytes and macrophages predominate in the inflammatory infiltrate of infected placentas [8], [9]. Our findings point to MIG, MCP-1 and IP-10 as pivotal in recruiting such cells into the placenta. Little is known concerning IP-10 and MIG in the context of placental malaria, although IP-10 has been shown to be produced by cultured intervillous blood mononuclear cells isolated from the placentas of women infected with *P. falciparum* [30]. High levels of IP-10 are found in pre-eclampsia [31], and have recently been shown to be involved in the pathogenesis of cerebral malaria, both in mice [32] and in humans [33]. In the latter study IP-10 was identified as a biomarker associated with mortality in *P. falciparum-*mediated cerebral malaria. IP-10 has both pro- and anti-inflammatory properties, and has been proposed to be a potential link between inflammation and anti-angiogenesis in preeclampsia [34]. We found that IP-10 levels were increased irrespective of gravidity, emphasizing its association with infection, and lending support for this chemokine as a potential biomarker.

We also found increased levels of uPAR in the infected women compared to uninfected controls, although this did not reach statistical significance. Elevated levels of uPAR have previously been shown to predict LBW in maternal malaria [35], and to be associated with parasitaemia in children with acute *P. falciparum* infections [36]. Various immune cells express uPAR which can be shed from the cell surface [37]. Given the fact that activated monocytes have increased expression and release uPAR, it could be that monocytes within the placenta contribute to the high blood levels of this factor during malaria infection. In addition, high levels of pro-inflammatory cytokines, or presence of adherent and circulating parasitized erythrocytes could also contribute to enhanced uPAR release from vascular endothelial cells, but the exact source for this molecule is still not known.

Of all the molecules quantified, only the concentration of RANTES decreased upon infection. Low circulating levels of RANTES have previously been shown to be associated with severe malaria [38], and especially with mortality in children with cerebral malaria [39]. Thrombocytopenia is frequent in severe malaria cases and is associated with increased mortality in children [40]. Since platelets are a major reservoir of RANTES in the circulation [41], it has been suggested that lower levels of RANTES in patients with severe malaria may be due to parasite-induced thrombocytopenia [42]. Of relevance to our study is the fact that pregnant women with acute uncomplicated malaria become more thrombocytopenic than non-pregnant women [43]. The possible pathological relevance of the decreased amounts of RANTES in women with *P. falciparum* during pregnancy – all of whom, it should be stressed, were asymptomatic at the time of diagnosis – therefore remains to be clarified.

Increased levels of IL-10 at delivery in the infected women have been reported by Kabyemela and colleagues [44]. Here, our longitudinal study has extended that finding, revealing that IL-10, in tandem with IP-10 levels, increase in infected women irrespective of their gestational age. IL-10 is a key cytokine both in the protection and in the pathogenesis of malaria. High levels of IL-10 may be beneficial to the host by reducing inflammatory responses, but on the other hand may be detrimental by suppressing protective anti-parasitic Th1-type responses. Low levels of IL-10 or a low IL-10/TNF ratio are associated with malarial anemia in African children [45]. Elevated levels of IL-10 in infected pregnant women may, thus, plausibly play a role in the down-regulation of pathological parasite-induced Th1-type responses in order to maintain a healthy pregnancy. The anti-inflammatory properties of IL-10 are mediated through blockade of monocytes/macrophage functions including the production of pro-inflammatory cytokines such as IL-6, TNF and IL-1 [46]. The latter are primary mediators of acute phase responses that regulate the induction of acquired immune responses. The asymptomatic nature of the infections in our pregnant women could, thus, reflect the suppressive effects of increased amounts of IL-10. The increased IL-10 levels seen in asymptomatic pregnant Ghanaian women infected with *P. falciparum* in their third trimester further confirm of our observations [24]. The important role IL-10 plays in suppressing Th1 responses during pregnancy is reflected by the increased levels of IL-10 seen during normal healthy pregnancies compared to non-pregnant controls [47].

The combination of biomarkers may be a better way to provide better diagnostic or prognostic accuracy than single markers. In an attempt to identify the best biomarker(s) of *P. falciparum* infection during pregnancy from amongst the panel of molecules quantified, we first used a logistic regression model that revealed IL-10, IP-10, MIG and RANTES as potentially useful in this regard. We then used ROC curve analysis as the most appropriate means of determining predictive values. The conclusion from those analyses is that the combination of increased IL-10 and IP-10 with decreased RANTES levels was most predictive of infection. While the result of this study is promising, the specificity of this combination requires further detailed investigation in a larger sample that should optimally include women with symptomatic as well as asymptomatic *P. falciparum* infections and also needs to be validated in other populations with differing levels of malaria endemicity. In this context, it should be noted that, in the Benin cohort of the STOPPAM study, PCR-based detection of ‘occult’ infections with *P. falciparum* at inclusion – undetected by either RDT or microscopy - has revealed significant associations with elevated plasma IL-10 levels (N Tuikue Ndam, unpublished data).

One major limitation of this study is the relatively small sample size, but this lack of power is at least partly offset by our use of samples from two closely matched controls per case. Placental histological evidence of infections that could have shed further light on the issue was not available to us due to technical problems during placental biopsy preparations and storage. We did not assess all potential biomarkers, including some more recently identified, but the plasma samples are still available and, resources permitting, could easily be screened to identify other candidates.

### Conclusion

To the best of our knowledge, there are no comprehensive prospective, longitudinal studies that describe cytokine and chemokine profiles during pregnancy and at delivery in an African cohort. Our study shows that IL-10, IP-10 and RANTES are increased upon infection with *P. falciparum* and therefore might be valuable for diagnostic purposes during pregnancy-associated malaria. The biomarkers that we have identified will need to be validated together with other biomarkers that have recently been associated with placental infection alongside malaria rapid diagnostic tests and PCR to compare accuracy and whether they could be combined to improve PM diagnosis. Our results contribute to the overall picture of *P. falciparum*-induced changes in cytokine and chemokine levels during pregnancy but more detailed studies are needed to further clarify the mechanisms underlying the patho-physiology of pregnancy-associated malaria.

## References

[1] Steketee RW, Nahlen BL, Parise ME, Menendez C (2001) The burden of malaria in pregnancy in malaria-endemic areas. The American Journal of Tropical Medicine and Hygiene 64: 28–35. 10.4269/ajtmh.2001.64.28 11425175

[2] Fried M, Duffy PE (1996) Adherence of plasmodium falciparum to chondroitin sulfate A in the human placenta. Science (New York, N.Y.) 272: 1502–1504. 10.1126/science.272.5267.1502 8633247

[3] Menendez C, Ordi J, Ismail MR, Ventura PJ, Aponte JJ, et al (2000) The impact of placental malaria on gestational age and birth weight. The Journal of Infectious Diseases 181: 1740–1745. 10823776 10.1086/315449

[4] Dorman EK, Shulman CE, Kingdom J, Bulmer JN, Mwendwa J, et al (2002) Impaired uteroplacental blood flow in pregnancies complicated by falciparum malaria. Ultrasound in Obstetrics & Gynecology : The Official Journal of the International Society of Ultrasound in Obstetrics and Gynecology 19: 165–170. 10.1046/j.0960-7692.2001.00545.x 11876809

[5] Umbers AJ, Boeuf P, Clapham C, Stanisic DI, Baiwog F, et al (2011) Placental malaria-associated inflammation disturbs the insulin-like growth factor axis of fetal growth regulation. The Journal of Infectious Diseases 203: 561–569. 21216864 10.1093/infdis/jiq080 PMC3071224

[6] Crocker IP, Tanner OM, Myers JE, Bulmer JN, Walraven G, et al (2004) Syncytiotrophoblast degradation and the pathophysiology of the malaria-infected placenta. Placenta 25: 273–282. 15028419 10.1016/j.placenta.2003.09.010

[7] Conroy AL, Liles WC, Molyneux ME, Rogerson SJ, Kain KC (2011) Performance characteristics of combinations of host biomarkers to identify women with occult placental malaria: A case-control study from malawi. PloS One 6: e28540. 22174834 10.1371/journal.pone.0028540 PMC3236186

[8] Ordi J, Ismail MR, Ventura PJ, Kahigwa E, Hirt R, et al (1998) Massive chronic intervillositis of the placenta associated with malaria infection. The American Journal of Surgical Pathology 22: 1006–1011. 9706981 10.1097/00000478-199808000-00011

[9] Rogerson SJ, Pollina E, Getachew A, Tadesse E, Lema VM, et al (2003) Placental monocyte infiltrates in response to plasmodium falciparum malaria infection and their association with adverse pregnancy outcomes. The American Journal of Tropical Medicine and Hygiene 68: 115–119. 12556159

[10] Abrams ET, Brown H, Chensue SW, Turner GD, Tadesse E, et al (2003) Host response to malaria during pregnancy: Placental monocyte recruitment is associated with elevated beta chemokine expression. Journal of Immunology (Baltimore, Md. : 1950) 170: 2759–2764. 10.4049/jimmunol.170.5.2759 12594307

[11] Suguitan AL, Jr, Leke RG, Fouda G, Zhou A, Thuita L, et al (2003) Changes in the levels of chemokines and cytokines in the placentas of women with plasmodium falciparum malaria. The Journal of Infectious Diseases 188: 1074–1082. 14513430 10.1086/378500

[12] Fried M, Muga RO, Misore AO, Duffy PE (1998) Malaria elicits type 1 cytokines in the human placenta: IFN-gamma and TNF-alpha associated with pregnancy outcomes. Journal of Immunology (Baltimore, Md. : 1950) 160: 2523–2530. 9498798

[13] Moormann AM, Sullivan AD, Rochford RA, Chensue SW, Bock PJ, et al (1999) Malaria and pregnancy: Placental cytokine expression and its relationship to intrauterine growth retardation. The Journal of Infectious Diseases 180: 1987–1993. 10558956 10.1086/315135

[14] Fievet N, Moussa M, Tami G, Maubert B, Cot M, et al (2001) Plasmodium falciparum induces a Th1/Th2 disequilibrium, favoring the Th1-type pathway, in the human placenta. The Journal of Infectious Diseases 183: 1530–1534. 11319691 10.1086/320201

[15] Rogerson SJ, Brown HC, Pollina E, Abrams ET, Tadesse E, et al (2003) Placental tumor necrosis factor alpha but not gamma interferon is associated with placental malaria and low birth weight in malawian women. Infection and Immunity 71: 267–270. 12496175 10.1128/IAI.71.1.267-270.2003 PMC143363

[16] Brabin BJ, Johnson PM (2005) Placental malaria and pre-eclampsia through the looking glass backwards? Journal of Reproductive Immunology 65: 1–15. 15694963 10.1016/j.jri.2004.09.006

[17] Wegmann TG, Lin H, Guilbert L, Mosmann TR (1993) Bidirectional cytokine interactions in the maternal-fetal relationship: Is successful pregnancy a TH2 phenomenon? Immunology Today 14: 353–356. 8363725 10.1016/0167-5699(93)90235-D

[18] Raghupathy R (1997) Th1-type immunity is incompatible with successful pregnancy. Immunology Today 18: 478–482. 9357139 10.1016/s0167-5699(97)01127-4

[19] Thevenon AD, Zhou JA, Megnekou R, Ako S, Leke RG, et al (2010) Elevated levels of soluble TNF receptors 1 and 2 correlate with plasmodium falciparum parasitemia in pregnant women: Potential markers for malaria-associated inflammation. Journal of Immunology (Baltimore, Md. : 1950) 185: 7115–7122. 10.4049/jimmunol.1002293 PMC2988086 20980627

[20] Bodker R, Akida J, Shayo D, Kisinza W, Msangeni HA, et al (2003) Relationship between altitude and intensity of malaria transmission in the usambara mountains, tanzania. Journal of Medical Entomology 40: 706–717. 14596287 10.1603/0022-2585-40.5.706

[21] Mmbando BP, Vestergaard LS, Kitua AY, Lemnge MM, Theander TG, et al (2010) A progressive declining in the burden of malaria in north-eastern tanzania. Malaria Journal 9: 216. 20650014 10.1186/1475-2875-9-216 PMC2920289

[22] Huynh BT, Fievet N, Gbaguidi G, Borgella S, Mevo BG, et al (2011) Malaria associated symptoms in pregnant women followed-up in benin. Malaria Journal 10: 72. 21453493 10.1186/1475-2875-10-72 PMC3076273

[23] Mshana RN, Boulandi J, Mshana NM, Mayombo J, Mendome G (1991) Cytokines in the pathogenesis of malaria: Levels of IL-I beta, IL-4, IL-6, TNF-alpha and IFN-gamma in plasma of healthy individuals and malaria patients in a holoendemic area. Journal of Clinical & Laboratory Immunology 34: 131–139. 1667945

[24] Wilson NO, Bythwood T, Solomon W, Jolly P, Yatich N, et al.. (2010) Elevated levels of IL-10 and G-CSF associated with asymptomatic malaria in pregnant women. Infectious Diseases in Obstetrics and Gynecology 2010: 317430. Epub 2010 Jul 12. 10.1155/2010/317430 PMC2913525 20706538

[25] Silver KL, Conroy AL, Leke RG, Leke RJ, Gwanmesia P, et al (2011) Circulating soluble endoglin levels in pregnant women in cameroon and malawi-associations with placental malaria and fetal growth restriction. PloS One 6: e24985. 21966395 10.1371/journal.pone.0024985 PMC3178568

[26] Silver KL, Zhong K, Leke RG, Taylor DW, Kain KC (2010) Dysregulation of angiopoietins is associated with placental malaria and low birth weight. PloS One 5: e9481. 20208992 10.1371/journal.pone.0009481 PMC2830425

[27] Opsjln SL, Wathen NC, Tingulstad S, Wiedswang G, Sundan A, et al (1993) Tumor necrosis factor, interleukin-1, and interleukin-6 in normal human pregnancy. American Journal of Obstetrics and Gynecology 169: 397–404. 8362955 10.1016/0002-9378(93)90096-2

[28] Sharma A, Satyam A, Sharma JB (2007) Leptin, IL-10 and inflammatory markers (TNF-alpha, IL-6 and IL-8) in pre-eclamptic, normotensive pregnant and healthy non-pregnant women. American Journal of Reproductive Immunology (New York, N.Y.: 1989) 58: 21–30. 10.1111/j.1600-0897.2007.00486.x 17565544

[29] Melgarejo E, Medina MA, Sanchez-Jimenez F, Urdiales JL (2009) Monocyte chemoattractant protein-1: A key mediator in inflammatory processes. The International Journal of Biochemistry & Cell Biology 41: 998–1001. 18761421 10.1016/j.biocel.2008.07.018

[30] Chaisavaneeyakorn S, Moore JM, Otieno J, Chaiyaroj SC, Perkins DJ, et al (2002) Immunity to placental malaria. III. impairment of interleukin(IL)-12, not IL-18, and interferon-inducible protein-10 responses in the placental intervillous blood of human immunodeficiency virus/malaria-coinfected women. The Journal of Infectious Diseases 185: 127–131. 11756993 10.1086/338013

[31] Szarka A, Rigo J, Jr, Lazar L, Beko G, Molvarec A (2010) Circulating cytokines, chemokines and adhesion molecules in normal pregnancy and preeclampsia determined by multiplex suspension array. BMC Immunology 11: 59. 21126355 10.1186/1471-2172-11-59 PMC3014878

[32] Campanella GS, Tager AM, El Khoury JK, Thomas SY, Abrazinski TA, et al (2008) Chemokine receptor CXCR3 and its ligands CXCL9 and CXCL10 are required for the development of murine cerebral malaria. Proceedings of the National Academy of Sciences of the United States of America 105: 4814–4819. 18347328 10.1073/pnas.0801544105 PMC2290783

[33] Jain V, Armah HB, Tongren JE, Ned RM, Wilson NO, et al (2008) Plasma IP-10, apoptotic and angiogenic factors associated with fatal cerebral malaria in india. Malaria Journal 7: 83. 18489763 10.1186/1475-2875-7-83 PMC2405803

[34] Gotsch F, Romero R, Friel L, Kusanovic JP, Espinoza J, et al (2007) CXCL10/IP-10: A missing link between inflammation and anti-angiogenesis in preeclampsia? The Journal of Maternal-Fetal & Neonatal Medicine : The Official Journal of the European Association of Perinatal Medicine, the Federation of Asia and Oceania Perinatal Societies, the International Society of Perinatal Obstetricians 20: 777–792. 10.1080/14767050701483298 PMC2396489 17943641

[35] Ostrowski SR, Shulman CE, Peshu N, Staalsoe T, Hoyer-Hansen G, et al (2007) Elevated plasma urokinase receptor predicts low birth weight in maternal malaria. Parasite Immunology 29: 37–46. 17187653 10.1111/j.1365-3024.2006.00916.x

[36] Perch M, Kofoed P, Fischer TK, Co F, Rombo L, et al (2004) Serum levels of soluble urokinase plasminogen activator receptor is associated with parasitemia in children with acute plasmodium falciparum malaria infection. Parasite Immunology 26: 207–211. 15491469 10.1111/j.0141-9838.2004.00695.x

[37] Wilhelm OG, Wilhelm S, Escott GM, Lutz V, Magdolen V, et al (1999) Cellular glycosylphosphatidylinositol-specific phospholipase D regulates urokinase receptor shedding and cell surface expression. Journal of Cellular Physiology 180: 225–235. 10395292 10.1002/(SICI)1097-4652(199908)180:2<225::AID-JCP10>3.0.CO;2-2

[38] Ochiel DO, Awandare GA, Keller CC, Hittner JB, Kremsner PG, et al (2005) Differential regulation of beta-chemokines in children with plasmodium falciparum malaria. Infection and Immunity 73: 4190–4197. 15972509 10.1128/IAI.73.7.4190-4197.2005 PMC1168587

[39] John CC, Opika-Opoka R, Byarugaba J, Idro R, Boivin MJ (2006) Low levels of RANTES are associated with mortality in children with cerebral malaria. The Journal of Infectious Diseases 194: 837–845. 16941352 10.1086/506623

[40] Gerardin P, Rogier C, Ka AS, Jouvencel P, Brousse V, et al (2002) Prognostic value of thrombocytopenia in african children with falciparum malaria. The American Journal of Tropical Medicine and Hygiene 66: 686–691. 12224575 10.4269/ajtmh.2002.66.686

[41] Ellis M, al-Ramadi B, Hedstrom U, Frampton C, Alizadeh H, et al (2005) Significance of the CC chemokine RANTES in patients with haematological malignancy: Results from a prospective observational study. British Journal of Haematology 128: 482–489. 15686455 10.1111/j.1365-2141.2004.05350.x

[42] Were T, Hittner JB, Ouma C, Otieno RO, Orago AS, et al (2006) Suppression of RANTES in children with plasmodium falciparum malaria. Haematologica 91: 1396–1399. 17018392 PMC13402947

[43] Tan SO, McGready R, Zwang J, Pimanpanarak M, Sriprawat K, et al (2008) Thrombocytopaenia in pregnant women with malaria on the thai-burmese border. Malaria Journal 7: 209. 18922167 10.1186/1475-2875-7-209 PMC2579302

[44] Kabyemela ER, Muehlenbachs A, Fried M, Kurtis JD, Mutabingwa TK, et al (2008) Maternal peripheral blood level of IL-10 as a marker for inflammatory placental malaria. Malaria Journal 7: 26. 18230163 10.1186/1475-2875-7-26 PMC2265723

[45] Othoro C, Lal AA, Nahlen B, Koech D, Orago AS, et al (1999) A low interleukin-10 tumor necrosis factor-alpha ratio is associated with malaria anemia in children residing in a holoendemic malaria region in western kenya. The Journal of Infectious Diseases 179: 279–282. 9841855 10.1086/314548

[46] de Waal Malefyt R, Abrams J, Bennett B, Figdor CG, de Vries JE (1991) Interleukin 10(IL-10) inhibits cytokine synthesis by human monocytes: An autoregulatory role of IL-10 produced by monocytes. The Journal of Experimental Medicine 174: 1209–1220. 1940799 10.1084/jem.174.5.1209 PMC2119001

[47] Holmes VA, Wallace JM, Gilmore WS, McFaul P, Alexander HD (2003) Plasma levels of the immunomodulatory cytokine interleukin-10 during normal human pregnancy: A longitudinal study. Cytokine 21: 265–269. 12823999 10.1016/s1043-4666(03)00097-8

